# From Allostatic Load to Allostatic State—An Endogenous Sympathetic Strategy to Deal With Chronic Anxiety and Stress?

**DOI:** 10.3389/fnbeh.2019.00047

**Published:** 2019-03-21

**Authors:** Enrico Ullmann, Seth W. Perry, Julio Licinio, Ma-Li Wong, Eliyahu Dremencov, Evgenii L. Zavjalov, Oleg B. Shevelev, Nikita V. Khotskin, Galina V. Koncevaya, Anna S. Khotshkina, Mikhail P. Moshkin, Maxim S. Lapshin, Maria V. Komelkova, Inna V. Feklicheva, Olga B. Tseilikman, Olga P. Cherkasova, Kamaldeep S. Bhui, Edgar Jones, Clemens Kirschbaum, Stefan R. Bornstein, Vadim Tseilikman

**Affiliations:** ^1^Department of Medicine, Carl Gustav Carus, Technical University of Dresden, Dresden, Germany; ^2^Department of Child and Adolescent Psychiatry, Psychotherapy, and Psychosomatics, University of Leipzig, Leipzig, Germany; ^3^School of Medical Biology, South Ural State University, Chelyabinsk, Russia; ^4^College of Medicine, SUNY Upstate Medical University, Syracuse, NY, United States; ^5^Institute of Molecular Physiology and Genetics, Centre for Biosciences, Slovak Academy of Sciences, Bratislava, Slovakia; ^6^Biomedical Research Center, Institute of Experimental Endocrinology, Slovak Academy of Sciences, Bratislava, Slovakia; ^7^Institute of Cytology and Genetics, Siberian Branch of the Russian Academy of Science (RAS), Novosibirsk, Russia; ^8^Biophysics Laboratory, Institute of Laser Physics, Siberian Branch of the Russian Academy of Science, Novosibirsk, Russia; ^9^Centre for Psychiatry, Wolfson Institute of Preventive Medicine, Queen Mary University of London, London, United Kingdom; ^10^Institute of Psychiatry Psychology & Neuroscience, King's College London, London, United Kingdom; ^11^Department of Psychology, Biopsychology, Technical University of Dresden, Dresden, Germany; ^12^Faculty of Life Sciences & Medicine, Endocrinology and Diabetes, Kings College London, London, United Kingdom

**Keywords:** behavior, stress-diseases, allostasis/homeostasis, glutamate, PTSD (post-traumatic stress disorder)

## Abstract

The concepts of allostatic load and overload, i. e., a dramatic increase in the allostatic load that predisposes to disease, have been extensively described in the literature. Here, we show that rats engaging in active offensive response (AOR) behavioral strategies to chronic predator scent stress (PSS) display less anxiety behavior and lower plasma cortisol levels vs. rats engaging in passive defensive response (PDR) behavioral strategies to chronic PSS. In the same chronic PSS paradigm, AOR rats also have higher lactate and lower glutamate levels in amygdala but not in control-region hippocampus vs. PDR rats. The implications of these findings for regulation of allostatic and stress responses, and post-traumatic stress disorder (PTSD) are discussed.

## Introduction

Many physiologic and behavioral consequences of homeostatic adaptations during chronic stress, and differences between homeostatic and allostatic regulation—particularly as regards the limbic hypothalamic pituitary adrenal (LHPA) axis—remain undefined. Advancing knowledge in these areas has the potential to significantly increase our understanding and treatment of stress and stress-related disorders, such as post-traumatic stress disorder (PTSD).

Allostasis (an adaptive process that adjusts homeostasis after acute stress), allostatic load (the physiological “costs” of maintaining allostasis), and allostatic overload (an excessive and likely pathologic increase in allostatic load), have been described in the literature (Ramsay and Woods, 2014). Neuroendocrine compensation of chronic stress acts as a homeostatic physiological process that should improve health and prolong life (De Kloet et al., 1998). A reinterpretation of Selye's theory of General Adaptation Syndrome clarifies that stress mediators can have both damaging and protective effects (Mcewen, 2005). Biological changes that occur during the adaption and exhaustion stages are referred to as allostasis and allostatic overload; they include not only activation of the LHPA axis and its downstream effector pathways, but also changes in immune responses, cardiovascular and energy metabolism, and hypothalamus-mediated behavior (Korte et al., 2005). The paradigms of allostatic load and overload have been demonstrated in various human and animal models, and the glucocorticoid cortisol is implicated as one of four primary allostatic mediators (Seeman et al., 1997). An allostatic state is defined by chronic deviation of regulatory systems away from their normal state of operation, to establish a new set point (Koob and Le Moal, 2001).

The limbic system, including the hippocampus and amygdala, is evidenced to mediate many neurodevelopmental consequences of childhood abuse. The hippocampus and amygdala are both densely populated with glucocorticoid receptors (GR), and in adolescents with major depression, increased amygdala-hippocampal volume ratios were associated with increased anxiety-related indicators of allostatic load (Macmillan et al., 2003), perhaps due to stimulation of dendritic arborization and new spine formation on pyramidal cells (Morimoto et al., 1996). GR also localize on glial cells in the cerebral cortex, mainly the prefrontal cortex (PFC), which in contrast shows decreased gray matter volume after stress-related experiences (Hanson et al., 2010). The anterior cingulate cortex also contains a high density of GR (Sarrieau et al., 1986), and reduced anterior cingulate cortex volume is one of the most consistent findings in subjects with chronic mental stress. Moreover, increased cortisol concentration was seen in depressed subjects, whereas subjects with chronic mental strain showed decreased cortisol levels (Morris et al., 2012). Furthermore, in historically traumatized populations (i.e., Jewish people who were three generations removed from the Holocaust), overprotective parenting behavior was associated with higher hair-steroid concentrations and dampened LHPA axis activity compared to German and Russian-German control subjects who also recalled overprotective parenting (Ullmann et al., 2018). These findings suggest allostatic load and overload scenarios leading to new allostatic set-points, mediated by glucocorticoid signaling.

The LHPA axis plays a key role in the stress response, and adrenal weight and corticosterone (CORT) concentrations have been shown increased in chronically stressed mice, with higher stress levels associated with generally higher activity in a behavioral task (Schwabe et al., 2008). While physiological responses to acute stress events have been well-studied, we have limited understanding of the processes for restoring homeostasis in the context of chronic stress. In particular, our understanding of behavioral learning modes in long-term chronically-stressed animals remains limited. We propose an integrative view whereby allostatic states, i.e., prolonged regulatory system departure from its regular functioning level, may increase stress resistance by inducing a lower allostatic set-point in chronically stressed mammals compared to non-stressed mammals.

### Glutamate (Glu)—A Stress-Related Neurotransmitter

Glu is a major excitatory neurotransmitter, excess Glu causes damage and inflammation, and CORT acts directly via membrane-associated mineralocorticoid receptors (MR) and GR to cause Glu release (Mcewen, 2017). Activation of glutamatergic projections to limbic structures, such as the amygdala, and brainstem structures, such as the solitary nucleus, are implicated in the stress response (Mathew et al., 2001). Thus, higher mGluR5 (metabotropic glutamate receptor 5) and lower FKBP5 (Peptidyl-prolyl cis-trans isomerase FK Binding Protein 5) expression were observed in cortical regions of subjects with PTSD (Holmes et al., 2017). Dysfunctional Glu neurotransmission is a cardinal feature of stress-related psychiatric conditions, especially PTSD (Averill et al., 2017). In a rat model of predator stress with implications for PTSD, Glu receptors activate the sympathetic nervous system (SNS) (Adamec et al., 1999, 2010).

### Lactate (Lact)—An Endogenous Metabolite to Deal With Chronic Stress

Enhanced physical activity with increased glycolysis leading to higher Lact levels may play key roles in PTSD (Rogatzki et al., 2015). Downstream of glycolysis, Lact is produced as a byproduct of anaerobic respiration in oxygen-depleted muscle cells following heavy exertion. Increased glucose metabolism in the amygdala of depressed subjects, and the positive effects of exercise in subjects with fatigue suggest possible antidepressant effects of Lact (Mustian et al., 2017; Magistretti and Allaman, 2018). On the other hand, flashbacks, a cardinal symptom of PTSD, were precipitated in Vietnam veterans following the intravenous administration of sodium Lact (Rainey et al., 1987).

### Objectives

The aims of this study are to: (1) determine whether there are differences in allostatic states for dealing with chronic stress in a threat-induced paradigm, and (2) test whether greater physical activity [i.e., an active offensive response (AOR)] during chronic anxiety (predator stress) is accompanied by altered LHPA axis (CORT) signaling and increased anaerobic glycolysis (lactate) in the amygdala, as may occur in the brain under conditions of high metabolic demand (Riske et al., 2017) and/or hypoxia (Schurr and Rigor, 1998) consequent to higher physical activity.

We investigated whether rats engaging in active (offensive) stress responses after chronic predator stress would show decreased anxiety and plasma CORT, and decreased Glu and higher Lact in the amygdala, compared to rats engaging in passive (defensive) stress responses.

## Methods

Experiments were performed with 28 male Sprague-Dawley rats (3 months-old) weighing 240–260 g, from the specific pathogen-free (SPF) vivarium of the Institute of Cytology and Genetics (ICG), Siberian Branch of the Russian Academy of Science (SB-RAS) (Novosibirsk, Russia). Rats were housed in sibling pairs in standard ventilated cages (IVC BlueLine, Tecniplast, Italy). Water and granulated forage (Ssniff, Soest, Germany) were given *ad libitum*. Animals were kept in a 14 h light (2 a.m.−4 p.m.)−10 h dark (4 p.m.−2 a.m.) cycle, temperature (22–24°C), and relative humidity (40–50%) controlled environment. The behavioral testing was always initiated at the start of the dark cycle when rodents are most active.

For our experiments, we used the standard elevated plus maze (EPM) test apparatus TS0502-R3 (http://www.openscience.ru/index.php?page=ts&item=002) with the following dimensions: H of the closed branch = 0.3 m; H of the open branch = 0.01 m; length of the branch = 0.5 m; branch width = 0.14 m; height from the floor = 0.55 m. The animals were initially placed in the center of the EPM and were considered to have “entered” a branch when all 4 feet were within the branch. Their behavior was evaluated using the 3D animal tracking system “EthoStudio” (http://ethostudio.com/new/en/about/), and the collector of the behavior previously did not work with any rats of our groups.

All animal experiments conformed to the requirements of the Council for International Organizations of Medical Sciences (CIOMS) and the International Council for Laboratory Animal Science (ICLAS) as described in “International Guiding Principles for Biomedical Research Involving Animals” (Geneva, Switzerland, 1990). The handling of all animals was identical. The study protocol was approved by the Committee for Bioethics and Humane Treatment of Laboratory Animals at South Ural State University, Russia.

## Behavioral measurements

### Passive Defensive and Active Offensive Behavior to Predator Scent Stress (PSS)

Following exposure to the predator odor stimulus, the rat's phenotypic behavioral pattern was classified into one of two groups (Table 1): Active offensive response (AOR), i.e., rats exhibiting the “stimulus-response” behavior pattern, and passive defensive response (PDR) groups. For example, increased grooming behavior is a characteristic response to increased stress and anxiety in PDR rats (Estanislau, 2012), whereas higher activity levels, increased marking of territory (by urination), and dominant sexual behavior are characteristic responses to stress in AOR animals (Le Moene and Agmo, 2017). The control group was exposed to neutral odor. For our stress protocol, fresh odor source was produced daily from urine of one intact male cat and used it on the same day. Every day, 240 g of sawdust from a single manufacturer (Cats Best EkoPlus by J. RETTENMAIER & SÖHNE, GERMANY) was used to generate a one-day source of urine contaminated sawdust. After being collected from the cat tray, the urine contaminated sawdust was thoroughly mixed, put in a plastic container, stored at room temperature for 3–5 h before the experiment started, and separated into 10 × 20 g portions for ten cages (2 rats per cage). The animals were exposed to the odor every day between 1 and 2 p.m., as follows: in each rat cage 20 g of urine contaminated sawdust was placed in a Petri dish covered by clear nylon tissue for 10 min, and during that period their behavior was registered. A total of 14 cages (including the control group), each housing two rats, were studied per day. The cages with control animals got the clear sawdust without cat urine contamination. Animals were grouped by body weight. Repeated exposure to the PSS may be a more accurate model for PTSD than the single acute exposure approach, since it minimizes the effect of confounding factors, such as the concentration of pheromones in each individual urine scent exposure. Indeed, rats repeatedly exposed to the PSS showed some abnormalities which were not observed after the acute exposure, such as decreased plasma corticosterone levels and adrenal hypotrophy (Manukhina et al., 2018).

**Table 1:** Behavior of the rats during exposure to cat urine.

**Passive Defensive Response (PDR)**	**Control**	**Active Offensive Response (AOR)**
Grooming behavior	Animals move freely within the cage with neutral scent	Actively smelling the stimulus throughout the exposure; occasionally licking and/or biting the Petri dish where the urine is located
“Freezing” in one place for more than 10 s to a few minutes		
Escaping to the furthest corner of the cage, with head positioned furthest away from the source of the smell (Petri dish)		Attempting to tear the protective material (tear-resistant nylon stocking) to get at the source of the odor
Trying to hide under or behind another animal		Marking territory by urinating and/or “climbing” the stimulus—the rat sits on top of the Petri dish with the stimulus

### Anxiety Measurements—EPM Test

The PSS outcome was evaluated using the EPM test (Lapiz-Bluhm et al., 2008; Serova et al., 2014). This test is widely used for measuring anxiety-like behavior based on the natural aversion of rats for open and elevated areas, as well as on their natural, spontaneous exploratory behavior in novel environments. Variables recorded included time spent in open and closed arms of the maze, and the number of entries into the open and closed arms. The Anxiety Index (AI) was calculated as follows: AI = 1—[(seconds in open arms/seconds on maze) + (number of entries into open arms/number of all entries)]/2 (Cohen et al., 2008). Anxious animals spend less time in the open arms of the maze.

### Magnetic Resonance Spectroscopy (MRS)

Rat amygdala and hippocampal neurometabolites were measured on a horizontal tomograph with a magnetic field of 11.7 Tesla (Bruker, Biospec 117/16 USR, Germany). The rats were anesthetized with gas (Isoflurane; Baxter Healthcare Corp., Deerfield, IL) using a Univentor 400 Anesthesia Unit (Univentor, Zejtun, Malta). The tomograph table contained a water circuit that maintained a surface temperature of 30°C, to preserve animal body temperature during the test. A pneumatic respiration sensor (SA Instruments, Stony Brook, NY), placed under the lower body, controlled the depth of anesthesia.

**Figure 1:**
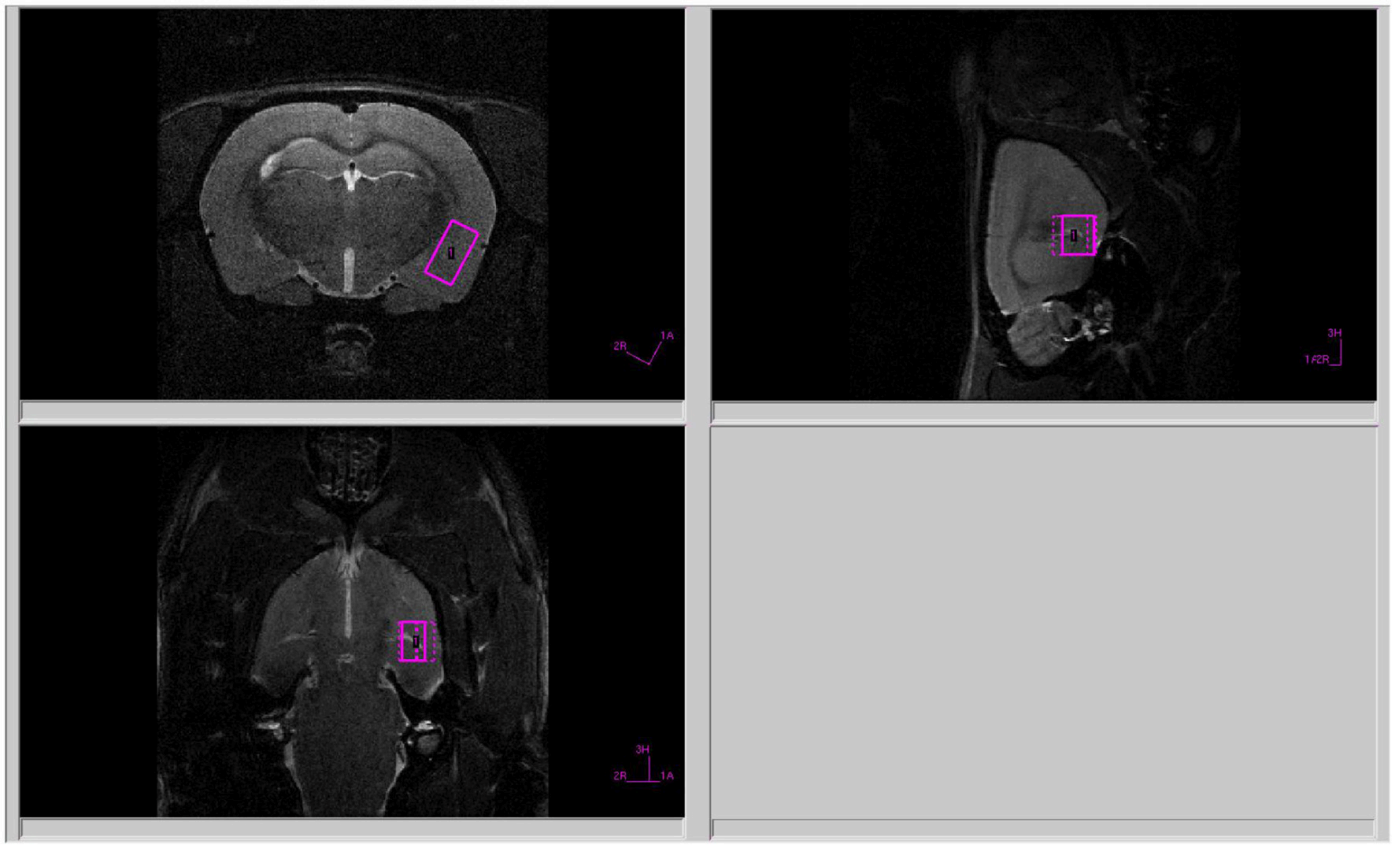
Voxel position during ^1^H MRS of the amygdala.

**Figure 2:**
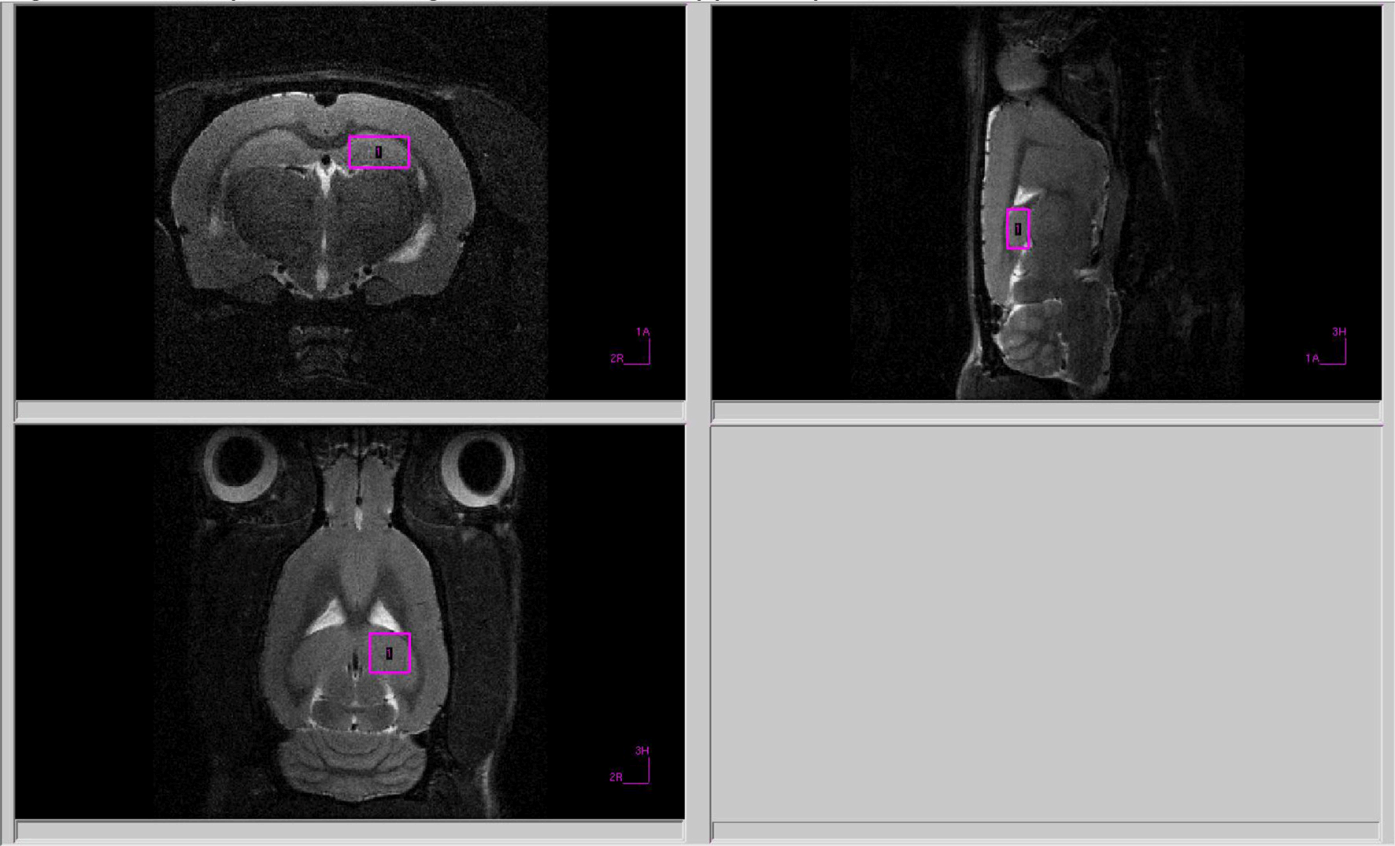
Voxel position during ^1^H MRS of the hippocampus.

All proton spectra of the rat amygdala and hippocampus were recorded with transmitter volume (T11232V3) and rat brain receiver surface (T11425V3) ^1^H radiofrequency coils (Bruker, Ettlingen, Germany). High-resolution T2-weighted images of the rat brain in three (axial, sagittal, and coronal) dimensions (section thickness, 0.5 mm; field of view, 2.5 × 2.5 cm for axial and 3.0 × 3.0 cm for sagittal and coronal sections respectively; matrix of 256 × 256 dots) were recorded by rapid acquisition with relaxation enhancement (TurboRARE), with the pulse sequence parameters TE = 11 ms, TR = 2.5 s for correct positioning of the spectroscopic voxels. Voxel dimensions were 3.0 × 1.5 × 3.0 mm for amygdala and 1.5 × 3.0 × 3.0 mm for the hippocampus. All voxels were manually placed according to a structural T2-weighted MRI images (Figures 1, 2). All proton spectra were recorded by spatially localized single-voxel stimulated echo acquisition mode (STEAM) spectroscopy with the pulse sequence parameters TE = 3 ms, TR = 5 s, 120 accumulations. Uniformity of the magnetic field was tuned within the selected voxel using FastMap (Gruetter, 1993) before each spectroscopic recording. The water signal was inhibited with a variable pulse power and optimized relaxation delays (VAPOR) sequence (Tkac et al., 1999).

### Processing of ^1^H Spectra

**Figure 3:**
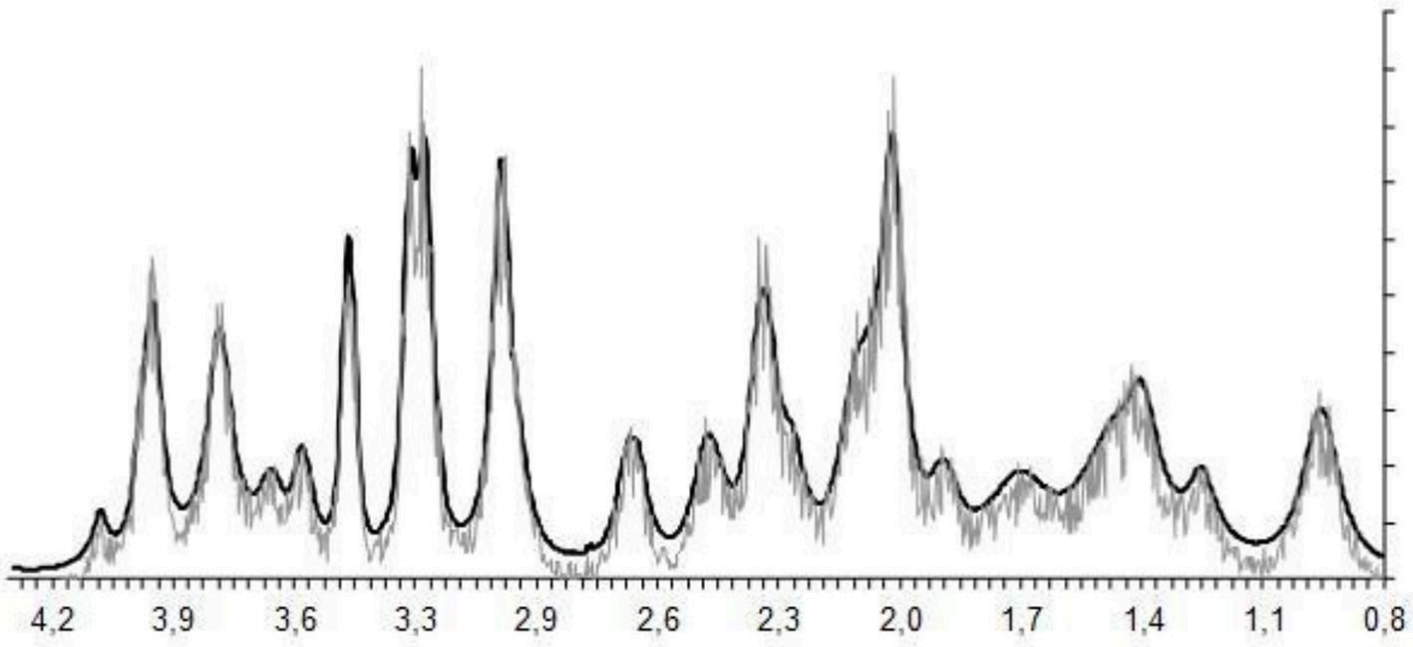
The process of spectral fitting and baseline correction.

The experimental ^1^H magnetic resonance spectra were processed, and the quantitative composition of metabolites was determined with a custom program similar to the LC Model software package (Provencher, 1993), which assume that the spectrum of a mixture of known compounds is a linear combination of analyzed components. The details of the data processing were published (Moshkin et al., 2014). The baseline correction is conducted automatically by the program in order to determine the spectral base-line for fitting of the spectrum obtained by ^1^H MRS. The process of fitting is presented on the real-time plot (Figure 3), and the fitting results data are stored in numerical form. The program capabilities allow the following 12 brain metabolites to be fitted to the MRS spectrum: N-acetylaspartate (NAA); phosphorylethanolamine (PEA); choline compounds (Cho); creatine + phosphocreatine (Cr + PCr); myo-inositol (mIno, Ins); alanine (Ala); lactate (Lac); glutamate + glutamine (Glu + Gln); aspartate (Asp); γ-aminobutyric acid (GABA); glycine (Gly); and taurine (Tau).

### Glucocorticoid Measurements

Between 11:00 a.m. and 1:00 p.m. on experimental day 28, rats were sacrificed by decapitation, and blood samples were collected in tubes with heparin. Blood samples were then centrifuged at 3,000 rpm for 15 min at +4°C. Plasma samples were aliquoted and stored in a −80°C freezer until use. After thawing, plasma CORT concentrations were measured by ELISA (Cusabio ELISA Kit, Texas, USA) per manufacturer's instructions. The assay sensitivity was 0.25 ng/ml, and the intra- and inter-assay coefficients of variation were both < 5%.

### Experimental Protocol

To induce chronic stress rats were exposed to cat urine scent in a Petri dish with litter for 10 min daily for 10 days (20 rats were submitted to stress exposure; 8 control rats were exposed to a neutral scent). All procedures were performed between 1:00 and 2:00 p.m. During the scent exposure protocol, stress-related behavior was captured daily via web-camera. The timeline for modeling PSS, evaluating stress-related behavior and anxiety, and measuring of metabolites (CORT, Glu/Gln, Lact) in plasma and brain, was as follows:

Days 1–10: PSS
Days 11–22: Rest
Day 23: Elevated plus-maze test
Day 27: Amygdala metabolite measurement by MRS
Day 28: Euthanasia, harvest blood and organs.

### Data Analyses

Data were analyzed with STATISTICA 10.0 and MS Excel software. Quantitative data are presented as mean ± SEM. One-way ANOVA with Fisher-LSD *post-hoc* tests were used to compare all outcome measures between two groups (e.g., control vs. AOR; control vs. PDR; AOR vs. PDR). *p* < 0.05 was considered significant.

## Results

### Behavioral Strategies to Deal With Chronic Anxiety-Stress

Chronically stressed rats were divided into two phenotypes based on their behavior in anxiety-related situations, as described above. The first phenotype was labeled AOR, i.e., rats exhibiting the “stimulus-response” behavior pattern (*n* = 9). The second phenotype was labeled PDR, i.e., rats exhibiting the “spatial” stress response strategy (*n* = 11). There were significant differences between the mean freezing frequencies of PDR and AOR rats (5.16 ± 1.85 and 2.2 ± 0.85, mean ± SEM, respectively; *p* < 0.005).

The animals' anxiety levels were evaluated using the EPM test 14 days after PSS cessation in AOR and PDR PSS exposed groups and controls. There was a significant influence of behavioral phenotype in response to PSS on a relative number of branch entries [*F*_(2, 25)_ = 10.84, *p* < 0.001], exploring [*F*_(2, 25)_ = 14.3, *p* < 0.001], and time [*F*_(2, 25)_ = 21.04, *p* < 0.0001] in open arms (OA). PSS led to increased OA entries, OA exploring, and OA times in the AOR rats vs. the PDR rats or unstressed controls. However, exploring in OA was similar in both groups subjected to PSS, and there were no differences with control. Overall, the different behavioral phenotypes in response to PSS exposures were characterized by the differences in anxiety levels 14 days post PSS cessation. AOR rats exhibited lower anxiety levels at 14-days post PSS compared to PDR rats.

### Long-Term Consequences of PSS on Anxiety Levels (Figure 4A)

An animal's behavioral phenotype in response to PSS significantly influenced anxiety measurements by the EPM test [*F*_(2, 25)_ = 12.57, *p* < 0.005]. AOR rats had lower anxiety levels (as they spent more time in the open arms of the maze) compared with PDR rats (−0.79 ± 0.031 and −0.97 ± 0.014, mean ± SEM, respectively; *p* < 0.005;), as well as compared with control (0.98 ± 0.017, mean ± SEM, respectively; *p* < 0.005). The anxiety levels between control and PDR rats were not significantly different.

**Figure 4:**
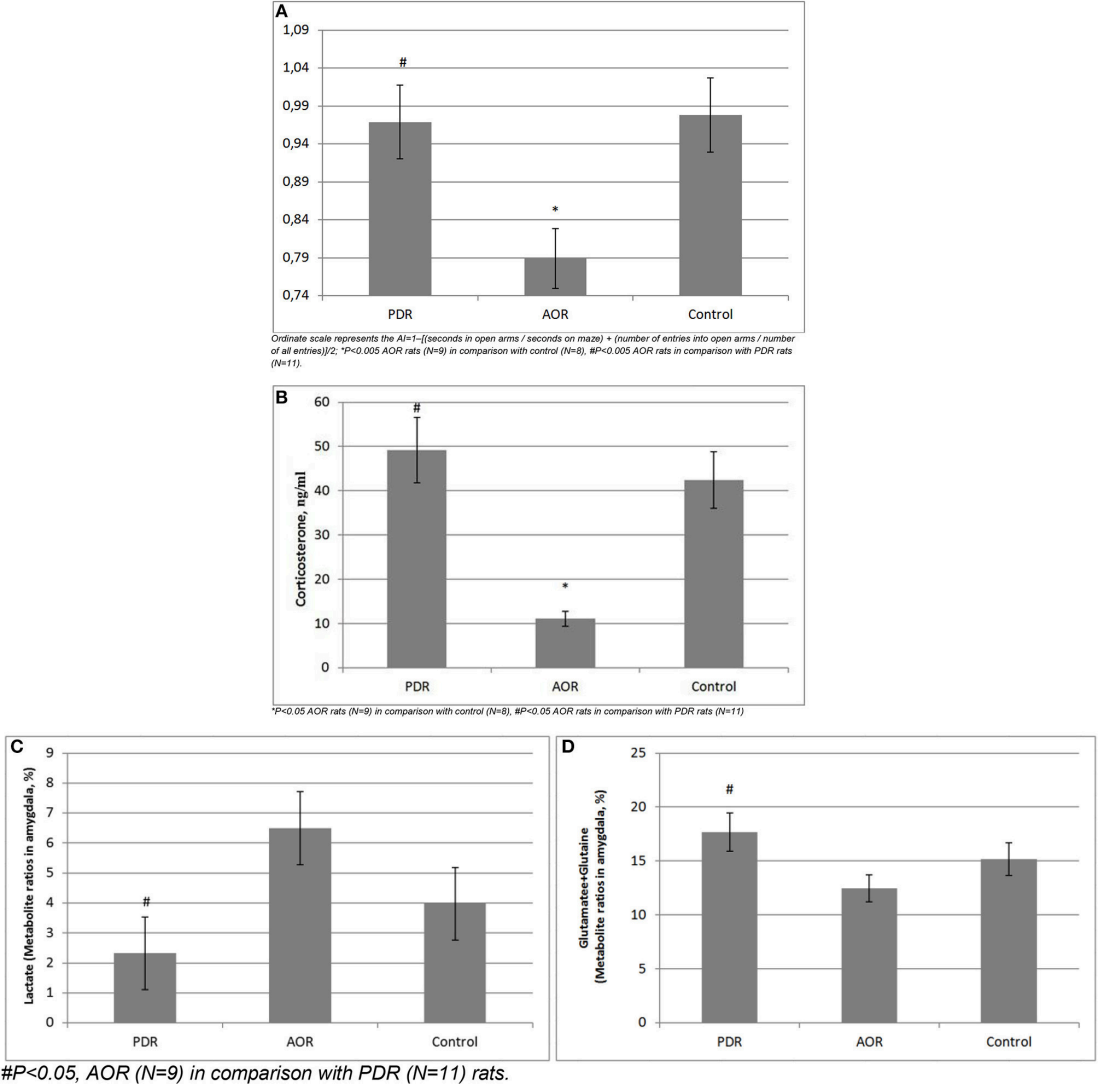
**(A)** Long-term consequences of PSS on anxiety index (AI) in chronically stressed rats. Ordinate scale represents the AI = 1–[(seconds in open arms/seconds on maze) + (number of entries into open arms/number of all entries)]/2; ^*^P < 0.005 AOR rats (*N* = 9) in comparison with control (*N* = 8), #*P* < 0.005 AOR rats in comparison with PDR rats (*N* = 11). **(B)** Long-term consequences of PSS on plasma CORT levels.^*^P < 0.05 AOR rats (*N* = 9) in comparison with control (*N* = 8), #*P* < 0.05 AOR rats in comparison with PDR rats (*N* = 11). **(C,D)** Long-term consequences of PSS on *in vivo* amygdala **(C)** Lactate and **(D)** Glutamate-Glutamine concentrations. #*P* < 0.05, AOR (*N* = 9) in comparison with PDR (*N* = 11) rats.

### Long-Term Consequences of PSS on Plasma Cort Levels (Figure 4B)

CORT levels in AOR rats (12.49 ng/ml ± 2.03, mean ± SEM) were decreased compared to control (42.4 ng/ml ± 9.46, mean ± SEM) and PDR (49.2 ng/ml ± 11.38, mean ± SEM) rats (*p* < 0.05 and *p* < 0.01, respectively). No significance differences in plasma CORT levels were found between control and PDR rats.

### Long-Term Consequences of PSS on Amygdala Metabolites *in vivo* (Figures 4C,D)

**Figure 5:**
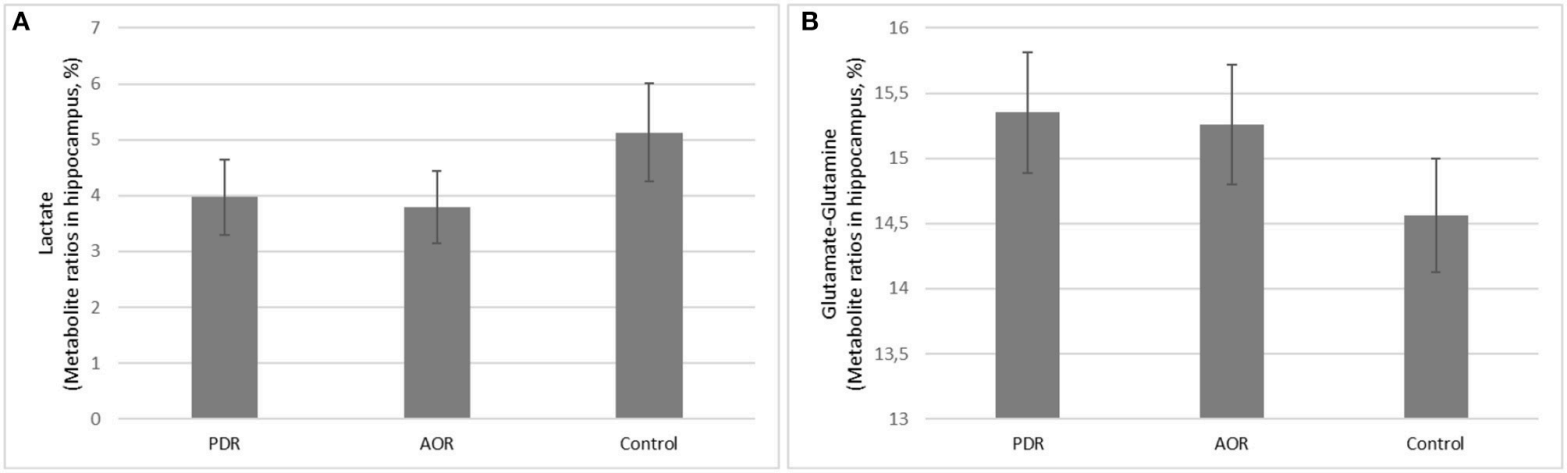
**(A,B)** Long-term consequences of PSS on *in vivo* hippocampus **(A)** Lactate and **(B)** Glutamate-Glutamine concentrations. **(A)** Lactate and **(B)** glutamate-glutamine were not significantly different between PDR (*N* = 11), AOR (*N* = 9), or control (*N* = 8) rats in control hippocampus region.

**Figure 6:**
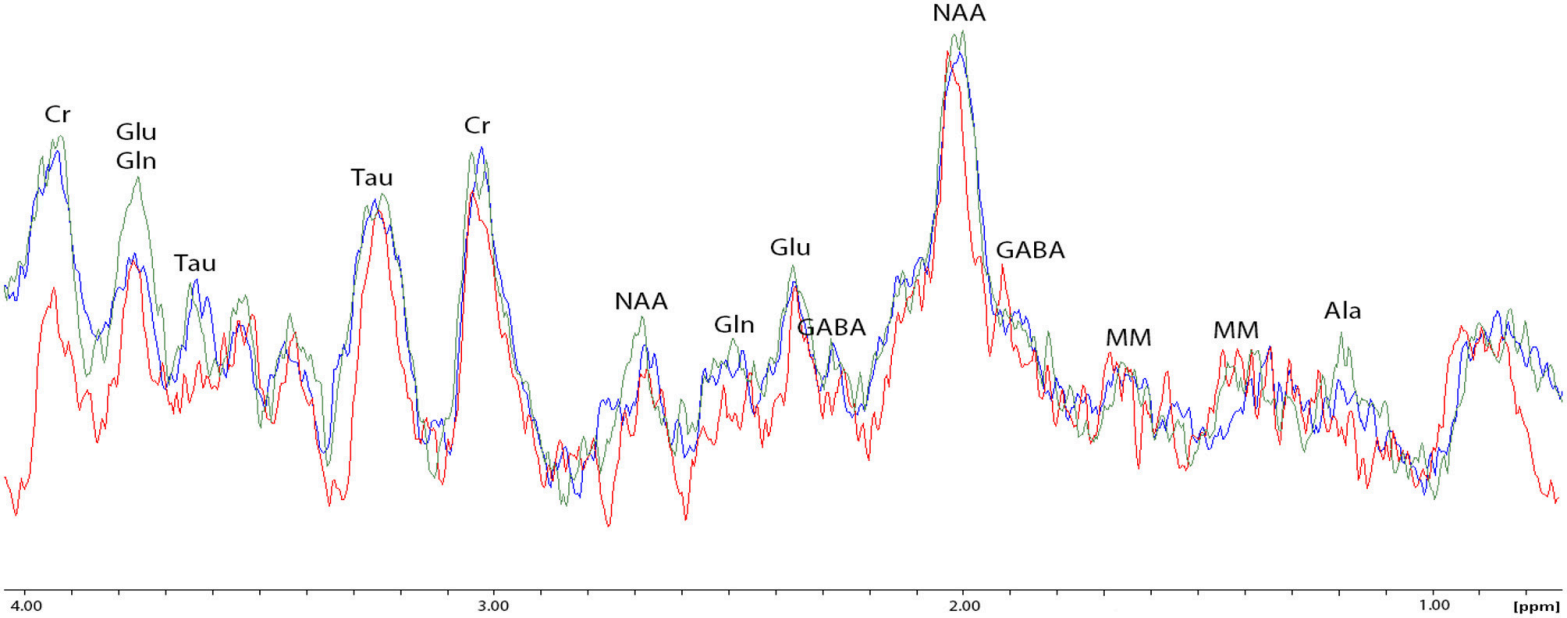
Spectral fitting (Proton-MRS) of metabolites in the amygdala of one representative rat from each of the three behavioral subtypes. NAA, N-acetylaspartate; PEA, phosphorylethanolamine; Cho, choline compounds; Cr, creatine; Ala, alanine; Lact, lactate; Glu + Gln, glutamate +glutamine; Asp, aspartate; GABA, γ-aminobutyric acid; Gly, glycine; and Tau, taurine. Red spectrum = PDR rat; blue spectrum = control rat (no chronic stress); green spectrum = AOR rat; ppm = parts per million; y-axis represents relative units in percentage; double peaks results from protons with different chemical environments.

Proton MRS was used to quantify amygdala metabolomics in control, AOR, and PDR rats after the final PSS exposure. There was a significant impact of behavioral phenotype on amygdala Lact concentrations [*F*_(2, 25)_ = 4.68, *p* < 0.05]. AOR rats exhibited increased Lact concentrations compared to PDR rats (5.81 ± 0.93 and 2.32 ± 0.76, mean ± SEM, respectively; *p* < 0.05). There was no significant difference in Lact concentrations between PDR and control (3.97 ± 0.98, mean ± SEM, respectively; *p* > 0.1) rats. Furthermore, there was significantly [*F*_(2, 25)_ = 3.65, *p* < 0.05] less mean Glu and Gln in AOR vs. PDR rats (13.41 ± 0.9 and 17.68 ± 1.42, mean ± SEM, respectively; *p* < 0.05). No significant differences in the concentrations of other metabolites were found between the groups (one-way ANOVA). Thus, as with the other parameters measured, the significant differences were found only in the AOR group (when compared to PDR or control groups). Time in OA in AOR rats was positively correlated with lactate level in the amygdala (*r* = 0.721, *p* = 0.024) and negatively correlated with plasma corticosterone level (*r* = −0.645, *p* = 0.032). Also, plasma corticosterone level was negatively correlated with lactate concentration in the amygdala (*r* = −0.721, *p* = 0.017). In the hippocampus, another limbic control structure, there were no significant differences in lactate or glutamate-glutamine between AOR and PDR, or between AOR/PDR and control (Figure 5). Spectral pictures of an AOR rat, a PDR rat, and a control rat are demonstrated in Figure 6.

## Discussion

This study demonstrated decreased CORT, Glu/Gln, and anxiety in AOR vs. PDR rats after chronic predator stress, suggesting a possible endogenous calming psychophysiological mechanism in the AOR phenotype. In addition, Lact levels were elevated in the amygdala of AOR compared to PDR animals, which would be consistent with increased metabolic demand (Riske et al., 2017) and/or hypoxia (Schurr and Rigor, 1998) as might be expected with a more “active” (AOR) vs. passive (PDR) behavioral phenotype. Interestingly, time in the open-arm of the maze (indicating less-anxious behavior) was positively correlated with lactate levels in the amygdala of AOR rats, suggesting a possible link between the behavioral phenotype and physiology. Such changes were not found in the hippocampus, suggesting this may be a local rather than global phenomena, and future studies in additional brain regions can shed further light on the regional vs. global nature of these findings.

The progression from homeostasis to allostatic overload has been well-established and includes hormonal, structural and epigenetic changes (Mathew et al., 2001; Macmillan et al., 2003; Morris et al., 2012; Klengel et al., 2013; Holmes et al., 2017). As evidence of increased dendritic remodeling in the hippocampus during allostatic load, it has been shown that dendritic remodeling can be blocked by phenytoin, which in turn inhibits Glu release and antagonizes sodium and likely T-type calcium channels that are activated during glutamate-induced excitation (Korte et al., 2005).

At first glance, our results showing decreased CORT levels in AOR rats may appear to conflict with those findings. However, the recurrent nature of the predator stress stimuli in our study suggests a switch from increased glucocorticoid levels in response to the acute stress, to an allostatic state with decreased glucocorticoid levels following continued predator stress. We propose these allostatic states may be described as: (1) an allostatic flight/fight response mechanism, and (2) an allostatic freezing/passive response mechanism. Others (Bowen et al., 2014) have referred to these adaption processes as “active and passive coping styles.” However, we suggest that the endogenous/biological nature of these adaption states are not adequately reflected by the term “coping styles,” as hormonal changes, dendritic remodeling, and gene methylation processes occur. The physiological changes that were seen within these two threat-induced behavior responses (AOR and PDR) allowed us to assess the allostatic load in behaviorally active vs. passive animals (whose stress responses require minimal physical or physiological exertion).

The amygdala is a glucocorticoid-responsive structure, lower CORT levels lead to lower amygdala activity, and glutamatergic neurons mediate amygdala excitation (Mcewen, 2017). We posit that lower Glu levels in the AOR animals may represent low level activity of glutamatergic neurons in the amygdala, whereas the higher Glu levels in PDR rats may reflect dendritic remodeling not only in the hippocampus (Mcewen et al., 2016) but also in the amygdala.

Reduced CORT appears to be associated with less freezing behavior (Takahashi and Rubin, 1993), and increased CORT is associated with freezing behavior in non-stressed rhesus monkeys (Kalin et al., 1998). Both those studies are consistent with our findings of lower CORT in AOR (i.e., non-freezing) rats, suggesting dampened LHPA axis responsiveness to fear. This physiological reaction may occur via gene methylation processes (Klengel et al., 2013; Holmes et al., 2017), although our changes in neurotransmitters (e.g., Glu/Gln) are also consistent with the time scale of the observed LHPA axis downregulation in AOR rats.

Historically, some have postulated that corticosteroids and the sympathomimetic amines have analogous roles because their multiple action sites and the nature of their induced responses are often similar (Ramey and Goldstein, 1957). However, while their roles are complementary and integrative, they are not interchangeable. Many actions of the sympathomimetic amines are not elicited in the absence of corticosteroids. Steroids maintain the integrity and responsiveness of tissues that are in the process of reacting to the sympathomimetic amines. This relationship is best seen on exposure to stress, when lower steroid levels may be elicited by heightened sympathetic-medullary activity. In the absence of corticosteroids, responses to neurohormones are diminished, and the deleterious effects of adrenal insufficiency are amplified.

Given the closely intertwined roles of the sympathomimetic amines and glucocorticoids, theoretically it would be conceivable that the glucocorticoids may also have roles analogous to sympathetic functions, and vice-versa the sympathomimetic amines may have roles analogous to parasympathetic functions. Mounting evidence in recent years suggests an interaction between the adrenal medulla and adrenal cortex, and an influence of adrenal innervations on adrenocortical functions (Ehrhart-Bornstein et al., 1998). Additionally, glutamatergic hippocampal giant mossy fiber terminals (MFTs) play a crucial role after chronic restraint stress, along with multiple interacting mediators of neuronal remodeling that include brain derived neurotrophic factor (BDNF) (Mcewen et al., 2016). Concerning cholinergic modulation of amygdala circuits in the formation and retention of fear memories (Jiang et al., 2016), we postulate that excitatory and inhibitory glutamatergic functions may exist in anxiety-related disorders.

Additionally, the effects of higher physical activity include increased glucose metabolism and, in extreme stress, increased lactate levels, which also ensure that brain and muscle metabolism can continue when glucose levels fall. Increased glucose metabolism in the amygdala of depressed subjects and the positive effect of exercise in subjects with fatigue suggest possible antidepressant effects of Lact (Mustian et al., 2017; Magistretti and Allaman, 2018). We found increased Lact levels in our AOR rats, as might be expected with this more “physically active” behavioral phenotype.

## Study Limitations and Future Directions

In this study, we found that rats demonstrating an AOR to chronic predator stress have less anxiety behavior, lower plasma corticosterone levels, and higher lactate and lower glutamate/glutamine levels in amygdala, compared to rats demonstrating a PDR to chronic predator stress. Integrating these results with supporting studies and existing literature, one possible explanation is that AOR rats enter an allostatic state in which adaptive behavioral responses serve to calm the animal and restore homeostasis following chronic fear and stress. However, our current data cannot exclude the possibility that animals stratify into AOR and PDR groups by their pre-existing underlying physiologies—i.e., perhaps AOR rats have “less anxious” physiology and neurochemistry/neurocircuitry. Therefore, follow-up studies will incorporate baseline reads for all animals, to help clarify whether the observed physiologic differences, suggestive of allostatic adaptive states, exist before or newly accompany the AOR vs. PDR behavioral phenotypes. These studies, together with appropriate blockade studies, will provide a clearer picture of which physiological changes may be causal vs. consequential to the AOR vs. PDR behavioral phenotypes. We also do not know and cannot predict the evolutionary or “survival” consequences of AOR vs. PDR phenotypes in a natural environment: perhaps AOR rats would be more likely to get eaten (which would make for a poor and evolutionarily short-lived adaptive strategy)! Finally, all measurements were done at the end of chronic stress exposure. Thus, our results reflect the end point, and while the amygdala and the hippocampus are critical limbic areas that regulate emotion, other brain areas are also likely involved in these observed behavioral paradigms (e.g., striatum, PFC) (Steimer, 2002) and will be investigated in future studies.

## Conclusions

Dealing with acute and chronic stress is an evolutionary challenge that affects all of us. Allostasis (from allostatic load to allostatic states) leads to decreased Glu/Gln levels, an effect also observed in our over-stressed AOR rats. Based on this and other data shown herein, we posit that allostasis may be a protective mechanism in rats for adapting to chronic stress, and further studies are warranted to expand these findings in rodents, and determine whether similar mechanisms exist for dealing with chronic stress in humans. Chronic stress affects not only our mental health and well-being, but also our physical health, and plays multiple roles in anxiety, depression, and other psychiatric disorders, as well as diabetes, cardiovascular, and neurologic diseases, and a range of inflammatory and metabolic conditions. The body and the brain have substantial capacity for adaptive plasticity; thus all changes described here are not necessarily irreversible (Korte et al., 2005). Our results provide new insights into the regulation of mammalian stress responses.

## Author Contributions

EU and VT organized the study, analyzed the data, drafted the manuscript, and prepared the figures and tables. EZ, OS, NK, GK, AK, MM, and OC collected the samples and analyzed the biomarkers in Novosibirsk. ML, MK, IF, and OT designed the work plan together with the corresponding author (EU) and the team leader (VT) and formulated the scientific questions. CK, SB, KB, EJ, SWP, JL, M-LW, and ED provided intellectual and scientific input, data interpretation. All authors reviewed the manuscript.

### Conflict of Interest Statement

The authors declare that the research was conducted in the absence of any commercial or financial relationships that could be construed as a potential conflict of interest.
